# THE IMPACT OF THE R3 PROGRAM ON SERVICE UTILIZATION, COSTS, AND QUALITY OF LIFE

**DOI:** 10.1093/geroni/igz038.2856

**Published:** 2019-11-08

**Authors:** Marc A Cohen, Jennifer Hefele, Edward Miller, Pamela Nadash, Natalie Shellito, Elizabeth Simpson

**Affiliations:** 1 University of Massachusetts Boston Gerontology Institute, LeadingAge LTSS Center @UMass Boston, Boston, Massachusetts, United States; 2 University of Massachusetts Boston, Boston, Massachusetts, United States

## Abstract

Relatively few housing with services evaluations employ rigorous multiple group quasi-experimental designs rather than simple before/after research designs. This study employs a pre-post experimental design with a comparison group to analyze whether the R3 program led to reductions in certain key utilization measures, including emergency department visits, inpatient hospitalizations, ambulance usage, and skilled nursing facility admissions, over 18 months. Also examined is whether resident quality of life indicators changed over the period. Data derive from assessments with 410 residents in four intervention sites and 227 residents in five comparison group sites. Also obtained was data on emergency department transfers from first responder services and Medicare fee-for-service data from the local quality improvement organization. Results suggest that the program has positive implications for service utilization, costs, and quality of life. Early detection and intervention inherent in the R3 program may improve resident quality of life while lowering service utilization and costs.

